# Parafermions in moiré minibands

**DOI:** 10.1038/s41467-025-57035-x

**Published:** 2025-02-19

**Authors:** Hui Liu, Raul Perea-Causin, Emil J. Bergholtz

**Affiliations:** Department of Physics, Stockholm University, AlbaNova University Center, Stockholm, Sweden

**Keywords:** Quantum Hall, Topological matter

## Abstract

Moiré materials provide a remarkably tunable platform for topological and strongly correlated quantum phases of matter. Very recently, the first Abelian fractional Chern insulators (FCIs) at zero magnetic field have been experimentally demonstrated, and it has been theoretically predicted that non-Abelian states with Majorana fermion excitations may be realized in the nearly dispersionless minibands of these systems. Here, we provide telltale evidence based on many-body exact diagonalization for the even more exotic possibility of moiré-based non-Abelian FCIs exhibiting Fibonacci parafermion excitations. In particular, we obtain low-energy quantum numbers, spectral flow, many-body Chern numbers, and entanglement spectra consistent with the ${{\mathbb{Z}}}_{3}$ Read–Rezayi parafermion phase in an exemplary moiré system with tunable quantum geometry. Our results hint towards the robustness of moiré-based parafermions and encourage the pursuit in moiré systems of these non-Abelian quasiparticles that are superior candidates for topological quantum computing.

## Abstract (web summary)

Parafermions hold promise as building blocks for topological quantum computers but have remained experimentally elusive. Here, the authors employ numerical calculations to demonstrate that parafermion excitations can emerge in partially filled moiré minibands of twisted multilayer graphene systems.

## Introduction

In the last years, moiré materials have been established as an accessible and highly tunable platform for exploring strongly correlated topological phases of matter^1–7^. Most notably, specific van der Waals heterostructures consisting of twisted layers of graphene or transition metal dichalcogenides have been theoretically predicted^8–15^ and experimentally observed^16–22^ to host fractional Chern insulators (FCIs)—lattice analogs of fractional quantum Hall (FQH) states that can exist in the absence of a magnetic field^23–33^. So far, the FCI states that have been unambiguously identified in experiments are topologically equivalent to Abelian hierachical FQH states^34–37^ hosting Abelian anyon excitations^38,39^. While the possibility of exploring anyon physics at zero magnetic field and moderate temperatures is already remarkable, the pursuit of non-Abelian quasiparticles—which should appear as elementary excitations in more exotic FCI states—constitutes a more ambitious and potentially rewarding venture with prospects for fault-tolerant topological quantum computation^40^.

Recent experimental signatures^41^, suggested to arise from non-Abelian topological order at moiré filling fractions with even denominator, have sparked a series of theoretical works addressing the stability of Moore–Read (MR) states in moiré systems^42–49^. Strengthening the theoretical evidence based on the ground state degeneracy, which can correspond to either the MR phase or charge density waves (CDWs), further analysis counting quasi-hole excitations in entanglement spectra has shown that MR states could indeed be stable in moiré materials^42,47,48^. Unfortunately, the braiding operations allowed by the emergent Majorana excitations in MR states cannot generate any arbitrary unitary transformation and would thus be insufficient for universal quantum computation^40^. This issue can, however, be overcome by exploiting parafermions with richer braiding statistics, which have been predicted to appear in FQH systems^50^, FQH–superconductor heterostructures^51,52^, and certain non-Hermitian systems^53^, although their experimental realization remains elusive. A natural step forward in the context of moiré FCIs is thus to search for Fibonacci parafermions appearing in the ${{\mathbb{Z}}}_{3}$ Read–Rezayi (RR) state^50^—which is characterized by the clustering of composite fermions into triplets, as opposed to the pairing in the MR phase. Realizing parafermion excitations in the widely accessible and tunable moiré materials would open an exceptionally promising avenue in the development of fault-tolerant topological quantum computers.

In this work, we provide numerical evidence showing that the non-Abelian RR FCI at 3/5 filling can be realized in a moiré system. In particular, we perform many-body exact diagonalization on an exemplary moiré flat band based on a double twisted bilayer graphene (dTBG) model with tunable quantum geometry. First, we obtain the expected number of degenerate ground states appearing at momenta fulfilling the generalized Haldane statistics. After that, we verify the persistence of the many-body energy gap upon insertion of magnetic flux (i.e. twisted boundary conditions) and find that, on average, each ground state has a many-body Chern number of 3/5. More strikingly, we show that the state counting in the low-energy sector of the particle-cut entanglement spectrum (PES) exactly matches the number of allowed quasi-hole excitations. In addition, we find that the RR phase is destroyed when the average quantum metric of the considered flat band approaches asymptotically the ideal value for the second Landau level (LL), highlighting both the usefulness and the limitations of this quantity as a heuristic indicator for the emergence of non-Abelian phases. Finally, we show that the RR phase is less stable at 2/5 filling but still present—as reflected in the quasi-particle excitations accessed through the hole entanglement spectrum—, signaling the robustness of moiré-based parafermion FCIs.

## Results

### Setup

We consider an exemplary model based on dTBG describing moiré minibands with tunable quantum geometry. Concretely, the single-particle valley- and spin-polarized Hamiltonian reads^54^

1 $${H}_{0}({{{\bf{r}}}})=\left(\begin{array}{cccc}{u}_{1}I&{D}^{{{\dagger}} }({{{\bf{r}}}})&0&0\\ D({{{\bf{r}}}})&{u}_{2}I&\gamma I&0\\ 0&\gamma I&{u}_{3}I&{D}^{{{\dagger}} }({{{\bf{r}}}})\\ 0&0&D({{{\bf{r}}}})&{u}_{4}I\end{array}\right),$$

where the 2 × 2 matrix ${D}_{11}({{{\bf{r}}}})={D}_{22}({{{\bf{r}}}})=-2i{k}_{\theta }^{-1}\bar{\partial }$, *D*_12_(**r**) = *D*_21_( − **r**) = *αU*(**r**) describes TBG in the chiral limit^55^, *γ* is the coupling between the two TBG sheets, and *u*_*i*_ is a layer/sublattice potential. Here, *k*_*θ*_ = 4*π*/3*a*_*m*_ (*a*_*m*_ is the moiré lattice constant), $\bar{\partial }=\frac{1}{2}({\partial }_{x}+i{\partial }_{y})$, and $U({{{\bf{r}}}})={e}^{-i{{{{\bf{q}}}}}_{1}\cdot {{{\bf{r}}}}}+{e}^{i\phi }{e}^{-i{{{{\bf{q}}}}}_{2}\cdot {{{\bf{r}}}}}+{e}^{-i\phi }{e}^{-i{{{{\bf{q}}}}}_{3}\cdot {{{\bf{r}}}}}$ with *ϕ* = 2*π*/3 and ${{{{\bf{q}}}}}_{n}={C}_{3}^{n}{{{{\bf{k}}}}}_{\theta }$. Throughout this work, we consider the first magic angle^55^, i.e. *α* ≈ 0.586, and set *u*_1_ = 0.1, *u*_2_ = *u*_3_ = *u*_4_ = 0 in order to obtain an isolated flat band.

In this setting, the system is characterized by two consecutive non-degenerate and nearly flat bands exhibiting equal Chern number ${{{\mathcal{C}}}}=1$ and becoming degenerate in the limit *γ* → *∞* (see Supplementary Note 1 and Supplementary Fig. 1). The upper band maintains an ideal quantum geometry^56^ ${{{\rm{tr}}}}[g({{{\bf{k}}}})]=| \Omega ({{{\bf{k}}}})|$ as *γ* varies, where *g*(**k**) and Ω(**k**) are the Fubini–Study (FS) metric and the Berry curvature, respectively. A definition of the FS metric is provided in the Methods section. We focus on the lower band, which has a tunable FS metric with the average $\chi=\frac{1}{2\pi }{\int}_{{{{\rm{BZ}}}}} \, {d}^{2}{{{\bf{k}}}}\,\,{{{\rm{tr}}}}[g({{{\bf{k}}}})]$ ranging from *χ* = 1 as *γ* → 0 to *χ* = 3 as *γ* → *∞* corresponding to the average quantum metric of the lowest and the first excited (second) LLs, respectively^57^. We note, however, that this correspondence generally demands additional conditions related to vortexability^54^. Motivated by the fact that the RR states are energetically more competitive in the second LL of FQH systems^50,58,59^, we employ *γ* as a tuning knob to explore the emergence of this non-Abelian phase in our model.

### Read–Rezayi parafermion states

**Fig. 1:**
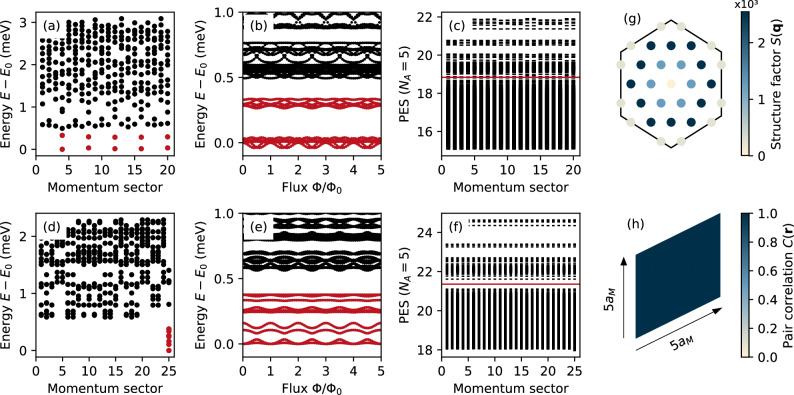
Evidence for moiré parafermions at *ν* = 3/5. **a** Low-lying many-body energy spectrum, (**b**) spectral flow, and (**c**) *N*_*A*_ = 5 particle-cut entanglement spectrum for a *N*_s_ = 20-site system with *γ* = 3.75. The corresponding results for *N*_s_ = 25 sites are shown in (**d**–**f**). The 10-fold nearly-degenerate ground states are marked by red dots. In the PES, the number of states below the red solid line is 14404 for *N*_s_ = 20 and 51255 for *N*_s_ = 25, both matching the quasi-hole excitation counting for Read--Rezayi states detailed in Supplementary Note 2. **g** Structure factor in the mini Brillouin zone and (**h**) pair-correlation function in the real-space unit cell for the Read–Rezayi ground states with *N*_s_ = 25. The spanning vectors for the considered systems are **T**_1_ = (3, 4) and **T**_2_ = ( − 2, 4) for *N*_s_ = 20, and **T**_1_ = (5, 0) and **T**_2_ = (0, 5) for *N*_s_ = 25.

We start by considering a fractionally filled dTBG with *ν* = 3/5 and a relatively large coupling *γ* = 3.75 between the two TBG sheets. Performing exact diagonalization on systems with *N*_s_ = 20 and *N*_s_ = 25 moiré sites (see Methods), we obtain 10 nearly-degenerate ground states separated from the excited states by an energy gap, cf. red dots in Fig. 1a, d. The splitting of these states is generically expected on the relatively small finite size systems accessible to exact diagonalization, but should go away in the large system limit. Moreover, we find that the ground states do not flow into excited states upon flux insertion (i.e. twisted boundary conditions), confirming the presence of a many-body gap, cf. Fig. 1b, e. The 10-fold ground state degeneracy is in agreement with that expected in the thin-torus limit, where a quantum Hall system is adiabatically mapped into a 1D lattice that can be exactly solved^60^. In particular, the RR ground states at filling *ν* = *k*/(*kM* + 2) are formed by *k* particles occupying *kM* + 2 consecutive sites and every two particles being separated by at least *M* sites^50,61,62^. Note that the 1D lattice sites in the thin torus are analogous to momentum points^23^. For fermionic Read–Rezayi states with *k* = 3 and *M* = 1, i.e., *ν* = 3/5, two distinct configurations are then expected, namely 1110011100 ⋯   and 1101011010 ⋯   (expressed in the occupation number representation), which together with their translation-invariant partners result in a 10-fold degenerate ground state. Furthermore, the calculated ground states in Fig. 1(a), (d) appear at center-of-mass momenta matching those expected from the aforementioned thin-torus configurations.

While the approximate ground state degeneracy and the spectral flow are consistent with the ${{\mathbb{Z}}}_{3}$ RR phase, they could also correspond to weakly entangled states such as CDWs—as has been shown for candidate MR states^47^. Thus, more information is needed to conclusively determine whether the ground states in Fig. 1a, d correspond to RR states. An additional aspect pointing towards the RR phase is the many-body Chern number, which we calculate to be 3/5 for each ground state on average. We take one step further and obtain unambiguous evidence by computing the PES of the ground states^23,63^. The PES, which is distinct from the entanglement entropy and provides much richer information, is obtained by dividing the many-body system into *A* and *B* subsystems consisting of *N*_*A*_ and *N*_*B*_ = *N*_e_ − *N*_*A*_ particles, and then calculating the eigenvalues of $-\log {\rho }_{A}$, where ${\rho }_{A}={{{{\rm{tr}}}}}_{B}[\,\frac{1}{{N}_{{{{\rm{d}}}}}}\,\mathop{\sum }_{i=1}^{{N}_{{{{\rm{d}}}}}}\,| {\Psi }_{i}\left.\right\rangle \left\langle \right.{\Psi }_{i}| ]$ is the reduced density matrix of *A*. Here, *ρ*_*A*_ carries crucial information about the quasi-hole excitations in the *N*_d_-fold degenerate ground states $| {\Psi }_{i}\left.\right\rangle$, which are characteristically distinct for Abelian FCIs, non-Abelian FCIs, and CDWs. In particular, a gap in the PES is expected, below which the number of states exactly matches the amount of quasi-hole excitations allowed by the specific quantum phase of the system.

In Fig. 1c, f we show the PES with *N*_*A*_ = 5 for the two considered system sizes. In addition, we mark with a red line the entanglement energy below which the number of states matches exactly the amount of allowed quasi-hole excitations (the analytical counting is detailed in Supplementary Note 2). For the smaller system (*N*_s_ = 20), the dense low-energy sector is followed by a region of sparsely distributed states resembling a gap (around the red line in Fig. 1c), below which the state counting is similar to that expected for the RR phase. Remarkably, the PES for the larger system (*N*_s_ = 25) shows a clear entanglement gap with the low-energy state counting being exactly equal to the number of quasi-hole excitations in the RR phase. This observation serves as smoking-gun evidence that the considered system is in the ${{\mathbb{Z}}}_{3}$ RR phase.

To further clarify the nature of the phase, we calculate the structure factor *S*(**q**) and pair-correlation function *C*(**r**) averaged over the tenfold quasi-degenerate ground states, which provide insights on the crystalline or liquid character of the system (see Methods). For liquid phases, *C*(**r**) approaches a constant for large **r**, while it shows a periodic arrangement of charges for CDWs. As shown in Fig. 1g, h, both the structure factor and the pair-correlation function indicate a liquid behavior—concretely, no prominent symmetry-breaking peaks appear in *S*(**q**) and *C*(**r**) is constant.

### Stability of parafermion FCIs and quantum geometry

**Fig. 2:**
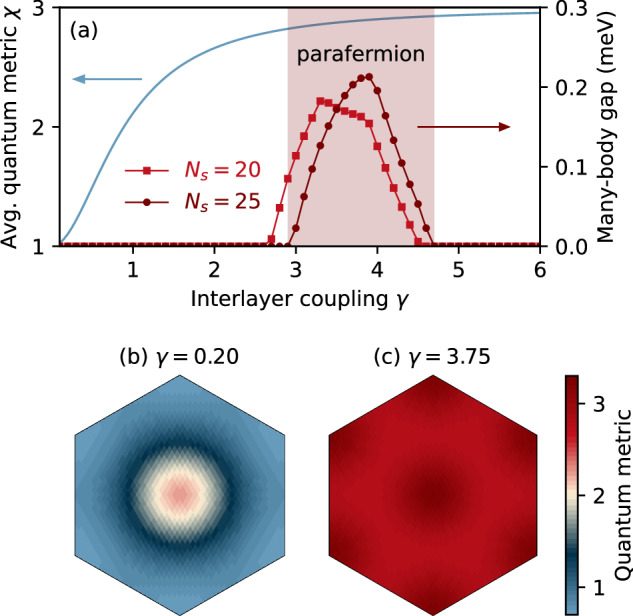
Stability of the parafermion phase and quantum geometry. **a** The many-body energy spectrum gap for non-Abelian ${{\mathbb{Z}}}_{3}$ RR states at *ν* = 3/5 (red dotted lines, right axis) as a function of the coupling strength *γ* between twisted layers is shown for system sizes *N*_s_ = 20 (squares) and *N*_s_ = 25 (circles) together with the averaged quantum metric *χ* (blue line, left axis). The energy gap is defined as the difference between the energies of the lowest non-RR state and the highest RR state (typically the 11th and the 10th lowest energies), and it is set to zero if the difference is negative. The shaded area denotes the regime where parafermions are stable. **b** Quantum metric *g*(**k**)*A*_BZ_/2*π* of the single-particle band for *γ* = 0.20 and (**c**) *γ* = 3.75. *A*_BZ_ is the Brillouin zone area.

Having demonstrated the existence of non-Abelian RR states, we now explore the stability of such topological phases in the parameter space associated with the quantum geometry, which serves as a heuristic indicator of the analogy between the partially filled moiré band and the LLs in two-dimensional electron gases^49,64–68^, although we note again that an accurate correspondence generally demands additional conditions^54^. For the *n*-th LL, the quantum metric and the Berry curvature satisfy^57^ ${{{\rm{tr}}}}[g({{{\bf{k}}}})]=(2n+1)| \Omega ({{{\bf{k}}}})|$, with *n* = 0, 1, 2…. Motivated by this property of LLs, it has been recently shown that both the Laughlin-like zero-energy modes^10^ and non-Abelian MR state can be realized in a moiré flat band with a (nearly) ideal quantum geometry^42–46,48^. As mentioned before, the average quantum metric *χ* of the considered flat band in dTBG increases gradually from 1 to 3 as *γ* is increased from 0 to infinity (see blue line in Fig. 2(a)). The parameter *γ* then provides an ideal tuning knob to study the transition between the physics of bands with quantum geometric properties similar to the lowest LL and the second LL.

In the strong coupling limit, *γ* → *∞*, the targeted band acquires an ideal average quantum metric *χ* = 3 and the band dispersion becomes exactly flat as in the second LL. However, while the RR states are energetically competitive in the first excited Landau level^58,59^, the many-body low-lying energy spectrum gap shown in Fig. 2a indicates their absence as *γ* → *∞*. Instead, the ${{\mathbb{Z}}}_{3}$ RR phase appears at an intermediate coupling strength, ranging from *γ* ≈ 3 to 4.5. We note that the fluctuation of the quantum metric is minimized around this range of *γ* (cf. Fig. 2b, c) and increases slightly for larger *γ* values (see Supplementary Fig. 2). These findings strongly suggest that the quantum geometry criterion is not sufficient to diagnose the non-Abelian topological phases. It nevertheless gives some intuition of where exotic states may occur—the RR states do in fact form in a region where the quantum geometry resembles that of the first excited rather than that of the lowest LL. We note that there are alternative ways of making moiré minibands that are close but not identical to the first excited LL such as considering other magic angles^69^, adding a “negative” magnetic field^70^ or periodic strain^71^, and considering bands further away from charge neutrality^42,43,46,48^.

In the weak coupling limit *γ* → 0, where the system approaches to two decoupled chiral TBG sheets, the average quantum metric *χ* → 1 shown in Fig. 2a and the quantum geometry distribution shown in Fig. 2b and Supplementary Fig. 2 suggest that the targeted flat band resembles the lowest LL, where Abelian hierarchy states at band filling *ν* = 3/5 and its particle-hole partner *ν* = 2/5^35–37^ are indeed present (see Supplementary Note 3).

### Particle-hole asymmetry and hole entanglement spectrum

**Fig. 3:**
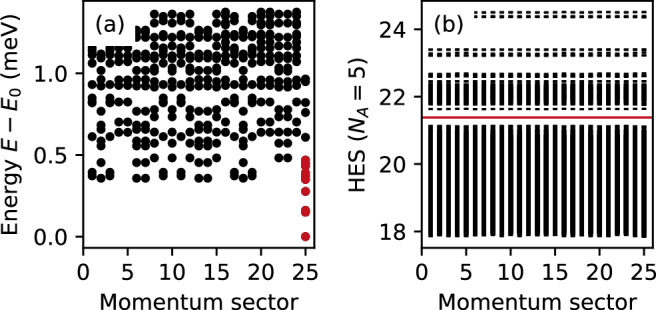
Moiré parafermions and competing states at *ν* = 2/5. **a** Low-lying energy spectrum at band filling *ν* = 2/5 and (**b**) the respective hole entanglement spectrum (HES) for *γ* = 3.75 in a system with size *N*_s_ = 25. In the HES, the number of states below the first entanglement gap (red line) is 51255, matching the quasi-particle counting of RR states at hole-filling 3/5.

We now search for the possibility of RR states at electronic band filling *ν* = 2/5, which in ideal LLs correspond to the particle-hole conjugate of the *ν* = 3/5 RR phase. It has been shown that, in general, particle-hole symmetry is broken in FCIs and moiré systems^8,72^—a fact that can be seen as a consequence of a fluctuating quantum metric^73,74^. In line with this asymmetry, we do not find a clear many-body energy gap of the RR states for filling *ν* = 2/5 at the system sizes considered, suggesting that the RR phase is less stable for *ν* = 2/5 than for *ν* = 3/5 (see Fig. 3a). In addition, although the tenfold quasi-degenerate states are still present at the correct momentum sector, their corresponding PES does not show a gap with the correct quasi-hole counting (see Supplementary Note 4 and Supplementary Fig. 5). This, however, should not be surprising: since the *ν* = 2/5 RR phase can be understood as the RR phase for holes at 3/5 hole-filling, adding holes to the system directly increases the ground-state energy as the 4-body interaction can no longer be minimized.

Instead, the *ν* = 2/5 RR phase allows a certain number of quasi-particle excitations without increasing the ground-state energy. This is reflected in the hole entanglement spectrum (HES), where the many-body system is divided into *A* and *B* subsystems with *N*_*A*_ and *N*_*B*_ = *N*_s_ − *N*_e_ − *N*_*A*_ now denoting the number of holes. Importantly, the HES (Fig. 3b) displays an entanglement gap below which the number of states matches the quasi-particle counting of the 3/5 hole-filling (i.e. *ν* = 2/5) RR phase, indicating that the calculated states indeed correspond to the non-Abelian RR phase. From a topological perspective, this then implies that the nature of the RR states remains largely unaffected by the renormalization of the hole dispersion that is expected to arise from fluctuations in the quantum metric^72^, even though these fluctuations may alter the ground state energies as suggested by the absence of a gap in Fig. 3a.

## Discussion

In this work we have demonstrated the emergence of Fibonacci (${{\mathbb{Z}}}_{3}$) parafermions in an exemplary moiré system based on dTBG. While a quantitatively realistic material prediction would require a consideration of finite bandwidth^75^ and band mixing effects^76–78^ as well as reliable estimates of system parameters (which in many cases remain controversial), our results are very encouraging for several reasons.

First, that an unscreened Coulomb interaction is sufficient to obtain parafermions is quite remarkable given that, for the simplest non-Abelian FCIs, fine-tuned interactions are necessary even in toy models^79,80^.

Second, although there is suggestive evidence for the possibility of RR parafermion quantum Hall states in Landau levels at *ν* = 12/5 and 13/5^58,59^, competing orders make the case less compelling than that of MR states at *ν* = 5/2. This situation may be opposite in the moiré context: at half filling of moiré bands charge density waves^15^ and the anomalous Hall crystal^81^ are additional fierce competitors with no direct LL analogues, while presumably no similarly competitive non-LL states exist at *ν* = 3/5 due to a lack of natural ordering patterns.

Third, bands with an average quantum geometry equal to that of excited Landau levels are not necessarily optimal for realizing RR parafermions. Figure 2 instead suggests that the most optimal bands have an average quantum geometry intermediate between the lowest and first excited Landau levels. It is quite likely that the optimal range of *χ* depends on the model/material and is related to the band flatness and quantum metric fluctuations. In this sense, the tunability of moiré bands offers enormous potential to achieve bands with the proper quantum geometric properties.

Fourth, although we find the ${{\mathbb{Z}}}_{3}$ RR parafermion states to be energetically favoured in a moderate parameter range (cf. Fig. 2a), it is striking that, even in cases when it is not apparent from the small system sizes avaliable whether the RR states will be competitive for a macroscopic number of particles, the entanglement spectrum shows telltale signatures of the RR states (cf. Fig. 3) suggesting that these states may prevail in a larger parameter range.

Finally, we emphasize that Fibonacci parafermions are arguably more desirable and elusive than Majorana fermions for fundamental reasons^82^: unlike the Majoranas, they are not possible to realize in effectively non-interacting Hermitian models^53^ and they potentially serve as building blocks for universal quantum computing^82^. These aspects have motivated the study of highly complex and technically extremely challenging setups such as FQH–superconductor heterostructures^51^. In contrast, moiré heterostructures offer a simple, versatile and tunable platform which does in principle not even require an external magnetic field.

In addition, we note that although the expected Hall conductivity for the RR phase is the same as for the Abelian hierarchy state at equal filling factor, many techniques proposed for quantum Hall systems could be imported to moiré systems in order to experimentally discern these two phases. In particular, the braiding statistics of parafermions can be probed via two-point contact interferometry^83–85^. Moreover, frequency-dependent shot noise measurements in Mach-Zehnder interferometers could provide access not only to the fractional charge (which is equal in the Abelian and ${{\mathbb{Z}}}_{3}$ RR parafermion phases) but also to the non-Abelian nature of parafermions^86^. Other proposals exploit non-linear *I* − *V* characteristics in tunneling experiments^87^ or Coulomb blockade in quantum dots^88,89^.

We hope that these observations will serve as inspiration for both the theoretical and experimental pursuit of parafermions emerging in moiré minibands.

## Methods

### Quantum metric of the single-particle band

The quantum geometric properties of the target single-particle band are determined by *ϕ*(**k**), i.e. the corresponding eigenstate of the momentum-space Hamiltonian, *H*_0_(**k**). Here, *H*_0_(**k**) is a matrix in the space of layers, sublattices, and the subbands that arise from zone-folding **k**-points outside of the moiré Brillouin zone up to a certain cutoff *N*_cutoff_. Each subband element is determined by moiré reciprocal lattice vectors **G** and ${{{{\bf{G}}}}}^{{\prime} }$ and corresponds to ${H}_{0}({{{\bf{k}}}}){| }_{{{{{\bf{GG}}}}}^{{\prime} }}=\int\,{{{{\rm{d}}}}}^{2}{{{\bf{r}}}}\,{{{{\rm{e}}}}}^{-i({{{\bf{k}}}}+{{{\bf{G}}}})\cdot {{{\bf{r}}}}}{H}_{0}({{{\bf{r}}}}){{{{\rm{e}}}}}^{i({{{\bf{k}}}}+{{{{\bf{G}}}}}^{{\prime} })\cdot {{{\bf{r}}}}}$. The quantum (Fubini–Study) metric *g*_*ij*_(**k**) describes the distance (or form factors) between states with a small momentum difference. Concretely, it is the real part of the quantum geometric tensor ${{{{\mathcal{Q}}}}}_{ij}({{{\bf{k}}}})$, i.e. ${g}_{ij}({{{\bf{k}}}})={{{\rm{Re}}}}[{{{{\mathcal{Q}}}}}_{ij}({{{\bf{k}}}})]$ where ${{{{\mathcal{Q}}}}}_{ij}({{{\bf{k}}}})={\partial }_{i}{\phi }^{{{\dagger}} }({{{\bf{k}}}}){\partial }_{j}\phi ({{{\bf{k}}}})-[{\partial }_{i}{\phi }^{{{\dagger}} }({{{\bf{k}}}})\phi ({{{\bf{k}}}})][{\phi }^{{{\dagger}} }({{{\bf{k}}}}){\partial }_{j}\phi ({{{\bf{k}}}})]$ (${\partial }_{i}\equiv {\partial }_{{k}_{i}}$ with *i* = *x*, *y*).

### Exact diagonalization

In order to tackle the many-body problem of interacting electrons, we first diagonalize *H*_0_(**r**) in the basis of Bloch states and then project the electron-electron interactions onto the considered nearly-flat band, obtaining ${H}_{{{{\rm{int}}}}}=\frac{1}{2}{\sum }_{{{{{\bf{k}}}}}_{1}{{{{\bf{k}}}}}_{2}{{{{\bf{k}}}}}_{3}{{{{\bf{k}}}}}_{4}}{V}_{{{{{\bf{k}}}}}_{1}{{{{\bf{k}}}}}_{2}{{{{\bf{k}}}}}_{3}{{{{\bf{k}}}}}_{4}}{c}_{{{{{\bf{k}}}}}_{1}}^{{{\dagger}} }{c}_{{{{{\bf{k}}}}}_{2}}^{{{\dagger}} }{c}_{{{{{\bf{k}}}}}_{3}}{c}_{{{{{\bf{k}}}}}_{4}}$. Here, the matrix element ${V}_{{{{{\bf{k}}}}}_{1}{{{{\bf{k}}}}}_{2}{{{{\bf{k}}}}}_{3}{{{{\bf{k}}}}}_{4}}$ ensures momentum conservation and contains single-particle form factors^8,90^ as well as the 2D bare Coulomb potential $V({{{\bf{q}}}})=\frac{{e}_{0}^{2}}{2A\epsilon {\epsilon }_{0}| {{{\bf{q}}}}| }$ with the average dielectric constant of the system *ϵ* = 5^91^. Next, we obtain the low-lying energy spectrum of many-body states by exact diagonalization of the spin- and valley-polarized interaction Hamiltonian *H*_int_. In particular, we consider a system of *N*_e_ electrons in *N*_s_ moiré sites with the filling *ν* = *N*_e_/*N*_s_ = 3/5, 2/5. We ensure that the calculations are converged with respect to the number of moiré subbands, ${(2{N}_{{{{\rm{cutoff}}}}}+1)}^{2}$, and note that *N*_cutoff_ = 4 is sufficient. In addition, we note that calculations in finite-size systems yield results that deviate with respect to infinite systems due to finite size effects. Here, we have employed state of the art methods and considered systems as large as possible to minimize such uncertainties.

### Structure factor and pair correlation

The structure factor helps measure if the ground states exhibit a preferred charge order and is defined as $S({{{\bf{q}}}})=\frac{1}{{N}_{{{{\rm{e}}}}}^{2}}\langle \rho ({{{\bf{q}}}})\rho (-{{{\bf{q}}}})\rangle -{\delta }_{{{{\bf{q}}}},0},$ with *ρ*(**q**) being the density operator projected onto the targeted nearly flat band. On the other hand, the real space pair-correlation function reads $C({{{\bf{r}}}})=\frac{1}{{N}_{{{{\rm{e}}}}}^{2}}\langle n({{{\bf{r}}}})n({{{\bf{0}}}})\rangle$, where *n*(**r**) is the real-space density operator. CDW order that breaks the translation symmetry of the underlying moiré lattice is characterized by structure factor peaks at symmetry points of the Brillouin zone, whereas liquids are distinguished by the absence of such peaks. Symmetry-breaking CDW peaks in *S*(**q**) would correspond to a periodic modulation of the pair-correlation function with a periodicity different than that of the underlying moiré lattice.

## Supplementary information

Supplementary Information

Transparent Peer Review file

## Source data

Source Data

## Data Availability

The data generated in this study and presented in the article are provided in the Source Data file. Source data are provided with this paper.

## Code availability

The codes used to generate and analyse the data are available from the corresponding author upon request.

## References

[1] Cao, Y. et al. Unconventional superconductivity in magic-angle graphene superlattices. *Nature* **556**, 43–50 (2018). 29512651 10.1038/nature26160

[2] Wu, F., Lovorn, T., Tutuc, E., Martin, I. & MacDonald, A. H. Topological insulators in twisted transition metal dichalcogenide homobilayers. *Phys. Rev. Lett.* **122**, 086402 (2019). 30932597 10.1103/PhysRevLett.122.086402

[3] Zheng, Z. et al. Unconventional ferroelectricity in moiré heterostructures. *Nature* **588**, 71–76 (2020). 33230334 10.1038/s41586-020-2970-9

[4] Andrei, E. Y. & MacDonald, A. H. Graphene bilayers with a twist. *Nat. Mater.* **19**, 1265–1275 (2020). 33208935 10.1038/s41563-020-00840-0

[5] Li, T. et al. Quantum anomalous Hall effect from intertwined moiré bands. *Nature* **600**, 641–646 (2021). 34937897 10.1038/s41586-021-04171-1

[6] Andrei, E. Y. et al. The marvels of moiré materials. *Nat. Rev. Mater.* **6**, 201–206 (2021).

[7] Mak, K. F. & Shan, J. Semiconductor moiré materials. *Nat. Nanotechnol.* **17**, 686–695 (2022). 35836003 10.1038/s41565-022-01165-6

[8] Abouelkomsan, A., Liu, Z. & Bergholtz, E. J. Particle-hole duality, emergent Fermi liquids, and fractional Chern insulators in moiré flatbands. *Phys. Rev. Lett.* **124**, 106803 (2020). 32216386 10.1103/PhysRevLett.124.106803

[9] Repellin, C. & Senthil, T. Chern bands of twisted bilayer graphene: Fractional Chern insulators and spin phase transition. *Phys. Rev. Res.* **2**, 023238 (2020).

[10] Ledwith, P. J., Tarnopolsky, G., Khalaf, E. & Vishwanath, A. Fractional Chern insulator states in twisted bilayer graphene: An analytical approach. *Phys. Rev. Res.* **2**, 023237 (2020).

[11] Liu, Z., Abouelkomsan, A. & Bergholtz, E. J. Gate-tunable fractional Chern insulators in twisted double bilayer graphene. *Phys. Rev. Lett.* **126**, 026801 (2021). 33512191 10.1103/PhysRevLett.126.026801

[12] Li, H., Kumar, U., Sun, K. & Lin, S.-Z. Spontaneous fractional Chern insulators in transition metal dichalcogenide moiré superlattices. *Phys. Rev. Res.* **3**, L032070 (2021).

[13] Devakul, T., Crépel, V., Zhang, Y. & Fu, L. Magic in twisted transition metal dichalcogenide bilayers. *Nat. Commun.* **12**, 6730 (2021). 34795273 10.1038/s41467-021-27042-9 PMC8602625

[14] Crépel, V. & Fu, L. Anomalous Hall metal and fractional Chern insulator in twisted transition metal dichalcogenides. *Phys. Rev. B* **107**, L201109 (2023).

[15] Wilhelm, P., Lang, T. C. & Läuchli, A. M. Interplay of fractional Chern insulator and charge density wave phases in twisted bilayer graphene. *Phys. Rev. B* **103**, 125406 (2021).

[16] Xie, Y. et al. Fractional Chern insulators in magic-angle twisted bilayer graphene. *Nature* **600**, 439–443 (2021). 34912084 10.1038/s41586-021-04002-3 PMC8674130

[17] Park, H. et al. Observation of fractionally quantized anomalous Hall effect. *Nature* **622**, 74–79 (2023). 37591304 10.1038/s41586-023-06536-0

[18] Cai, J. et al. Signatures of fractional quantum anomalous Hall states in twisted MoTe_2_. *Nature* **622**, 63–68 (2023). 37315640 10.1038/s41586-023-06289-w

[19] Zeng, Y. et al. Thermodynamic evidence of fractional Chern insulator in moiré MoTe_2_. *Nature* **622**, 69–73 (2023). 37494955 10.1038/s41586-023-06452-3

[20] Lu, Z. et al. Fractional quantum anomalous Hall effect in multilayer graphene. *Nature* **626**, 759–764 (2024). 38383622 10.1038/s41586-023-07010-7

[21] Xu, F. et al. Observation of integer and fractional quantum anomalous Hall effects in twisted bilayer MoTe_2_. *Phys. Rev. X* **13**, 031037 (2023).

[22] Spanton, E. M. et al. Observation of fractional Chern insulators in a van der Waals heterostructure. *Science* **360**, 62–66 (2018). 29496958 10.1126/science.aan8458

[23] Regnault, N. & Bernevig, B. A. Fractional Chern insulator. *Phys. Rev. X* **1**, 021014 (2011).

[24] Liu, Z. & Bergholtz, E. J. Recent developments in fractional Chern insulators, in *Encyclopedia of Condensed Matter Physics (Second Edition)*, edited by T. Chakraborty 10.1016/B978-0-323-90800-9.00136-0 (Academic Press, Oxford, 2024) second edition ed., pp. 515–538

[25] Bergholtz, E. J. & Liu, Z. Topological flat band models and fractional Chern insulators. *Int. J. Mod. Phys. B* **27**, 1330017 (2013).

[26] Parameswaran, S. A., Roy, R. & Sondhi, S. L. Fractional quantum Hall physics in topological flat bands. *Comptes Rendus Phys.* **14**, 816–839 (2013).

[27] Kol, A. & Read, N. Fractional quantum Hall effect in a periodic potential. *Phys. Rev. B* **48**, 8890–8898 (1993). 10.1103/physrevb.48.8890 10007108

[28] Tang, E., Mei, J.-W. & Wen, X.-G. High-Temperature Fractional Quantum Hall States. *Phys. Rev. Lett.* **106**, 236802 (2011). 21770532 10.1103/PhysRevLett.106.236802

[29] Sun, K., Gu, Z., Katsura, H. & Das Sarma, S. Nearly flatbands with nontrivial topology. *Phys. Rev. Lett.* **106**, 236803 (2011). 21770533 10.1103/PhysRevLett.106.236803

[30] Neupert, T., Santos, L., Chamon, C. & Mudry, C. Fractional quantum Hall states at zero magnetic field. *Phys. Rev. Lett.* **106**, 236804 (2011). 21770534 10.1103/PhysRevLett.106.236804

[31] Sheng, D., Gu, Z.-C., Sun, K. & Sheng, L. Fractional quantum Hall effect in the absence of Landau levels. *Nat. Commun.* **2**, 1–5 (2011). 10.1038/ncomms1380 PMC3160145 21750543

[32] Möller, G. & Cooper, N. R. Composite fermion theory for bosonic quantum Hall states on lattices. *Phys. Rev. Lett.* **103**, 105303 (2009). 19792327 10.1103/PhysRevLett.103.105303

[33] Kapit, E. & Mueller, E. Exact parent Hamiltonian for the quantum Hall states in a lattice. *Phys. Rev. Lett.* **105**, 215303 (2010). 21231318 10.1103/PhysRevLett.105.215303

[34] Laughlin, R. B. Anomalous quantum Hall effect: An incompressible quantum fluid with fractionally charged excitations. *Phys. Rev. Lett.* **50**, 1395–1398 (1983).

[35] Haldane, F. D. M. Fractional quantization of the Hall effect: A hierarchy of incompressible quantum fluid states. *Phys. Rev. Lett.* **51**, 605–608 (1983).

[36] Halperin, B. I. Statistics of quasiparticles and the hierarchy of fractional quantized Hall states. *Phys. Rev. Lett.* **52**, 1583–1586 (1984).

[37] Jain, J. K. Composite-fermion approach for the fractional quantum Hall effect. *Phys. Rev. Lett.* **63**, 199–202 (1989). 10040805 10.1103/PhysRevLett.63.199

[38] Leinaas, J. & Myrheim, J. On the theory of identical particles. *Il Nuovo Cim.* **37**, 132 (1977).

[39] Wilczek, F. Quantum mechanics of fractional-spin particles. *Phys. Rev. Lett.* **49**, 957–959 (1982).

[40] Nayak, C., Simon, S. H., Stern, A., Freedman, M. & Das Sarma, S. Non-Abelian anyons and topological quantum computation. *Rev. Mod. Phys.* **80**, 1083–1159 (2008).

[41] Kang, K. et al. Evidence of the fractional quantum spin Hall effect in moiré MoTe_2_. *Nature* **628**, 522–526 (2024). 38509375 10.1038/s41586-024-07214-5

[42] Reddy, A. P., Paul, N., Abouelkomsan, A. & Fu, L. Non-Abelian fractionalization in topological minibands. *Phys. Rev. Lett.* **133**, 166503 (2024). 39485960 10.1103/PhysRevLett.133.166503

[43] Wang, C. et al. Higher Landau-level analogues and signatures of non-Abelian states in twisted bilayer MoTe_2_, *preprint at* https://arxiv.org/abs/2404.05697 (2024). 10.1103/PhysRevLett.134.076503 40053999

[44] Zhang, L. & Song, X.-Y. Moore–Read state in half-filled moiré Chern band from three-body pseudopotential. *Phys. Rev. B* **109**, 245128 (2024).

[45] Xu, C., Mao, N., Zeng, T. & Zhang, Y. Multiple Chern bands in twisted MoTe_2_ and possible non-Abelian states, *preprint at* https://arxiv.org/abs/2403.17003 (2024). 10.1103/PhysRevLett.134.066601 40021170

[46] Ahn, C.-E., Lee, W., Yananose, K., Kim, Y. & Cho, G. Y. Non-Abelian fractional quantum anomalous Hall states and first Landau level physics of the second moiré band of twisted bilayer MoTe_2_. *Phys. Rev. B* **110**, L161109 (2024).

[47] Liu, H., Liu, Z. & Bergholtz, E. J. Non-Abelian fractional Chern insulators and competing states in flat moiré bands, *preprint at* https://arxiv.org/abs/2405.08887 (2024). 10.1103/43nq-ntqm 40981573

[48] Chen, F., Luo, W.-W., Zhu, W. & Sheng, D. Robust non-Abelian even-denominator fractional Chern insulator in twisted bilayer MoTe_2_, *preprint at* https://arxiv.org/abs/2405.08386 (2024). 10.1038/s41467-025-57326-3 PMC11876646 40032855

[49] Liu, Z., Mera, B., Fujimoto, M., Ozawa, T. & Wang, J. Theory of generalized Landau levels and implication for non-Abelian states, *preprint at* https://arxiv.org/abs/2405.14479 (2024).

[50] Read, N. & Rezayi, E. Beyond paired quantum Hall states: Parafermions and incompressible states in the first excited Landau level. *Phys. Rev. B* **59**, 8084–8092 (1999).

[51] Mong, R. S. K. et al. Universal topological quantum computation from a superconductor-Abelian quantum Hall heterostructure. *Phys. Rev. X* **4**, 011036 (2014).

[52] Liang, J., Simion, G. & Lyanda-Geller, Y. Parafermions, induced edge states, and domain walls in fractional quantum Hall effect spin transitions. *Phys. Rev. B* **100**, 075155 (2019).

[53] Fendley, P. Free parafermions. *J. Phys. A: Math. Theor.* **47**, 075001 (2014).

[54] Fujimoto, M. et al. Higher vortexability: zero field realization of higher Landau levels, *preprint at* https://arxiv.org/abs/2403.00856 (2024). 10.1103/PhysRevLett.134.106502 40153662

[55] Tarnopolsky, G., Kruchkov, A. J. & Vishwanath, A. Origin of magic angles in twisted bilayer graphene. *Phys. Rev. Lett.* **122**, 106405 (2019). 30932657 10.1103/PhysRevLett.122.106405

[56] Roy, R. Band geometry of fractional topological insulators. *Phys. Rev. B* **90**, 165139 (2014).

[57] Ozawa, T. & Mera, B. Relations between topology and the quantum metric for Chern insulators. *Phys. Rev. B* **104**, 045103 (2021).

[58] Rezayi, E. H. & Read, N. Non-Abelian quantized Hall states of electrons at filling factors 12/5 and 13/5 in the first excited Landau level. *Phys. Rev. B* **79**, 075306 (2009).

[59] Bonderson, P., Feiguin, A. E., Möller, G. & Slingerland, J. K. Competing topological orders in the ν = 12/5 quantum Hall state. *Phys. Rev. Lett.* **108**, 036806 (2012). 22400774 10.1103/PhysRevLett.108.036806

[60] Bergholtz, E. J. & Karlhede, A. Half-filled lowest Landau level on a thin torus. *Phys. Rev. Lett.* **94**, 026802 (2005). 15698208 10.1103/PhysRevLett.94.026802

[61] Ardonne, E., Bergholtz, E. J., Kailasvuori, J. & Wikberg, E. Degeneracy of non-Abelian quantum Hall states on the torus: domain walls and conformal field theory. *J. Stat. Mech. Theory Exp.* **2008**, P04016 (2008).

[62] Bernevig, B. A. & Regnault, N. Emergent many-body translational symmetries of Abelian and non-Abelian fractionally filled topological insulators. *Phys. Rev. B* **85**, 075128 (2012).

[63] Sterdyniak, A., Regnault, N. & Bernevig, B. A. Extracting excitations from model state entanglement. *Phys. Rev. Lett.* **106**, 100405 (2011). 21469776 10.1103/PhysRevLett.106.100405

[64] Wang, J. & Liu, Z. Hierarchy of ideal flatbands in chiral twisted multilayer graphene models. *Phys. Rev. Lett.* **128**, 176403 (2022). 35570419 10.1103/PhysRevLett.128.176403

[65] Wang, J., Cano, J., Millis, A. J., Liu, Z. & Yang, B. Exact Landau level description of geometry and interaction in a flatband. *Phys. Rev. Lett.* **127**, 246403 (2021). 34951815 10.1103/PhysRevLett.127.246403

[66] Varjas, D., Abouelkomsan, A., Yang, K. & Bergholtz, E. J. Topological lattice models with constant Berry curvature. *SciPost Phys.* **12**, 118 (2022).

[67] Goerbig, M. O. From fractional Chern insulators to a fractional quantum spin Hall effect. *Eur. Phys. J. B* **85**, 15 (2012).

[68] Claassen, M., Lee, C. H., Thomale, R., Qi, X.-L. & Devereaux, T. P. Position-momentum duality and fractional quantum Hall effect in Chern insulators. *Phys. Rev. Lett.* **114**, 236802 (2015). 26196819 10.1103/PhysRevLett.114.236802

[69] Wang, J., Zheng, Y., Millis, A. J. & Cano, J. Chiral approximation to twisted bilayer graphene: Exact intravalley inversion symmetry, nodal structure, and implications for higher magic angles. *Phys. Rev. Res.* **3**, 023155 (2021).

[70] Kolář, Kcv., Yang, K., von Oppen, F. & Mora, C. Hofstadter spectrum of Chern bands in twisted transition metal dichalcogenides. *Phys. Rev. B* **110**, 115114 (2024).

[71] Wan, X., Sarkar, S., Lin, S.-Z. & Sun, K. Topological exact flat bands in two-dimensional materials under periodic strain. *Phys. Rev. Lett.* **130**, 216401 (2023). 37295089 10.1103/PhysRevLett.130.216401

[72] Läuchli, A. M., Liu, Z., Bergholtz, E. J. & Moessner, R. Hierarchy of fractional Chern insulators and competing compressible states. *Phys. Rev. Lett.* **111**, 126802 (2013). 24093288 10.1103/PhysRevLett.111.126802

[73] Abouelkomsan, A., Yang, K. & Bergholtz, E. J. Quantum metric induced phases in moiré materials. *Phys. Rev. Res.* **5**, L012015 (2023).

[74] Liu, H., Yang, K., Abouelkomsan, A., Liu, Z. & Bergholtz, E. J. Broken symmetry in ideal Chern bands, *preprint at* https://arxiv.org/abs/2402.04303 (2024).

[75] Reddy, A. P. & Fu, L. Toward a global phase diagram of the fractional quantum anomalous Hall effect. *Phys. Rev. B* **108**, 245159 (2023).

[76] Xu, C., Li, J., Xu, Y., Bi, Z. & Zhang, Y. Maximally localized Wannier functions, interaction models, and fractional quantum anomalous Hall effect in twisted bilayer MoTe_2_, *Proc. Natl. Acad. Sci.* **121**, 10.1073/pnas.2316749121 (2024). 10.1073/pnas.2316749121 PMC10895274 38349878

[77] Yu, J. et al. Fractional Chern insulators versus nonmagnetic states in twisted bilayer MoTe_2_. *Phys. Rev. B* **109**, 045147 (2024).

[78] Abouelkomsan, A., Reddy, A. P., Fu, L. & Bergholtz, E. J. Band mixing in the quantum anomalous Hall regime of twisted semiconductor bilayers. *Phys. Rev. B* **109**, L121107 (2024).

[79] Liu, Z., Bergholtz, E. J. & Kapit, E. Non-Abelian fractional Chern insulators from long-range interactions. *Phys. Rev. B* **88**, 205101 (2013).

[80] Wang, D., Liu, Z., Liu, W.-M., Cao, J. & Fan, H. Fermionic non-Abelian fractional Chern insulators from dipolar interactions. *Phys. Rev. B* **91**, 125138 (2015).

[81] Sheng, D. N., Reddy, A. P., Abouelkomsan, A., Bergholtz, E. J. & Fu, L. Quantum anomalous Hall crystal at fractional filling of moiré superlattices. *Phys. Rev. Lett.* **133**, 066601 (2024). 39178450 10.1103/PhysRevLett.133.066601

[82] Alicea, J. & Fendley, P. Topological phases with parafermions: Theory and blueprints. *Annu. Rev. Condens. Matter Phys.* **7**, 119–139 (2016).

[83] Bonderson, P., Shtengel, K. & Slingerland, J. K. Probing non-Abelian statistics with quasiparticle interferometry. *Phys. Rev. Lett.* **97**, 016401 (2006). 16907388 10.1103/PhysRevLett.97.016401

[84] Nakamura, J., Liang, S., Gardner, G. C. & Manfra, M. J. Direct observation of anyonic braiding statistics. *Nat. Phys.* **16**, 931–936 (2020). 10.1038/s41467-022-27958-w PMC8763912 35039497

[85] Nakamura, J., Liang, S., Gardner, G. C. & Manfra, M. J. Fabry-Pérot interferometry at the ν = 2/5 fractional quantum Hall state. *Phys. Rev. X* **13**, 041012 (2023).

[86] Feldman, D. E., Gefen, Y., Kitaev, A., Law, K. T. & Stern, A. Shot noise in an anyonic Mach-Zehnder interferometer. *Phys. Rev. B* **76**, 085333 (2007).

[87] Gurarie, V. & Rezayi, E. Parafermion statistics and quasihole excitations for the generalizations of the paired quantum Hall states. *Phys. Rev. B* **61**, 5473–5482 (2000).

[88] Ilan, R., Grosfeld, E. & Stern, A. Coulomb blockade as a probe for non-Abelian statistics in Read–Rezayi states. *Phys. Rev. Lett.* **100**, 086803 (2008). 18352649 10.1103/PhysRevLett.100.086803

[89] Georgiev, L. S. Thermopower and thermoelectric power factor of Z_*k*_ parafermion quantum dots. *Nucl. Phys. B* **899**, 289–311 (2015).

[90] Zhang, Y.-H., Mao, D., Cao, Y., Jarillo-Herrero, P. & Senthil, T. Nearly flat Chern bands in moiré superlattices. *Phys. Rev. B* **99**, 075127 (2019).

[91] Wu, F., Lovorn, T., Tutuc, E. & MacDonald, A. H. Hubbard model physics in transition metal dichalcogenide moiré bands. *Phys. Rev. Lett.* **121**, 026402 (2018). 30085734 10.1103/PhysRevLett.121.026402

