# Supplementary material for: Parafermions in moiré minibands
Source: Nat Commun. 2025 Feb 19;16:1770. doi: 10.1038/s41467-025-57035-x (PMC11839918; doi:10.1038/s41467-025-57035-x)
Supplement: Supplementary file 1 — Supplementary Information [file 41467_2025_57035_MOESM1_ESM.pdf]

Supplementary Information for
‘Parafermions in moiré minibands’

Hui Liu,[1*] Raul Perea-Causin,[1*] and Emil J. Bergholtz[1*]

[1]*Department of Physics, Stockholm University, AlbaNova University Center, 106 91 Stockholm, Sweden*

*Corresponding authors: hui.liu@fysik.su.se, raul.perea.causin@fysik.su.se, emil.bergholtz@fysik.su.se

Supplementary Note 1. Band structure and quantum geometry

The single-particle band structure of the dTBG model considered is shown in Supplementary Figure 1 for three different values of the interlayer coupling γ. In the limit $\gamma \to 0$, the targeted nearly-flat band with Chern number $C = 1$ approaches the lowest twofold degenerate state, while at $\gamma \to \infty$ it approaches the higher flat band, which also has $C = 1$. We observe that the maximum Berry curvature fluctuation has a minimum around $\gamma = 0.4$ and increases for larger γ, while the quantum metric fluctuation generally decreases when increasing γ and has a minimum at $\gamma \approx 4$ that is however not very pronounced, cf. Supplementary Figure 2.

Supplementary Note 2. Counting of quasi-hole excitations in Read–Rezayi states

Below, we explicitly explain how to count the number of allowed quasi-hole excitations [1] in Read–Rezayi states. Concretely, we consider the case $N_A = 5$ and $\nu = 3/5$ discussed in the main manuscript. Here, the categories of the quasi-hole excitation states can be divided into four groups:

- No strings of 11 nor 111. The allowed states are the combinations of five 10 and zeros, yielding $n_1 = N_\mathrm{s}(N_\mathrm{s} - 10 + 5 - 1)!/((N_\mathrm{s} - 10)!5!)$ excitations.
- One string of 11. The allowed states are the combinations of one 110, three 10, and zeros, that is $n_2 = N_\mathrm{s}(N_\mathrm{s} - 9 + 4 - 1)!((N_\mathrm{s} - 9)!3!)$.
- Two strings of 11. The allowed states are the combinations of two 0110, one 1, and zeros, which give $n_3 = N_\mathrm{s}(N_\mathrm{s} - 9 + 3 - 1)!((N_\mathrm{s} - 9)!2!)$.
- One string of 111. The allowed states are the combinations of one 0011100, two 1, and zeros, resulting in $n_4 = N_\mathrm{s}(N_\mathrm{s} - 9 + 3 - 1)!((N_\mathrm{s} - 9)!2!)$.

In total, we obtain $n = n_1 + n_2 + n_3 + n_4 = 51255$ for $N_\mathrm{s} = 25$.

Supplementary Note 3. Hierarchical states at weak coupling

As mentioned in the main manuscript, in the weak coupling limit $\gamma \to 0$ the system resembles two decoupled chiral TBG sheets with an average quantum geometry $\chi \to 1$, suggesting that the targeted flat band [see Supplementary Figure 1(a)] emulates the lowest Landau level. Here, the ground states at filling $\nu = 3/5$ and $\nu = 2/5$ are expected to be Abelian states of the FQH hierarchy [2–4]. Our calculations indeed show a five-fold degenerate ground state at the expected momentum sectors for hierarchical $\nu = 3/5$ and $\nu = 2/5$ states, with a gap that persists upon flux insertion (i.e. twisted boundary conditions), see Supplementary Figure 3. As opposed to the RR phase, here the most stable state (with the largest gap in the many-body energy spectrum) is that for $\nu = 2/5$, which is analogous to the hierarchical FQH state for electrons deriving from the parent $\nu = 1/3$ state. This observation is in line with the lower stability of highly-entangled hole RR states—in the present case of Abelian hierarchy states, the state corresponding to holes is that at $\nu = 3/5$, which has a smaller gap in the many-body energy spectrum and is thus less stable. We note, however, that we do not observe a clear entanglement gap in the PES, which complicates the unambiguous identification of this phase.

Supplementary Note 4. Additional data for Read–Rezayi states

In Supplementary Figure 4(a), we show the many-body energy spectrum for the dTBG system with $\gamma = 3.75$ at $\nu = 3/5$ filling for a system size $N_\mathrm{s} = 30$. In this larger system, we also obtain 10 quasi-degenerate ground states at the correct

momenta (marked in red), suggesting that the parafermion phase remains stable in the thermodynamic limit. We also show in Supplementary Figure 4(b) the PES for a different particle-cut than the one considered in the main text. Concretely, we consider $N_A = 6$ (and $N_s = 25$) and observe the absence of any significant entanglement gap at low energies, indicating the absence of CDW order and thus strengthening the identification of the parafermion phase based on the data shown in the main manuscript. For $N_A < 6$, we obtain the expected counting of quasi-hole excitations for $\mathbb{Z}_3$ RR states.

As mentioned in the main text, the parafermion phase at band filling $\nu = 2/5$ is less stable than at $\nu = 3/5$. We show the many-body energy spectrum in the main manuscript, which exhibits the absence of a gap. In Supplementary Figure 5(a), we demonstrate that the Read–Rezayi states (red dots) do not flow into other excited states upon flux insertion. We also show the particle-cut entanglement spectrum in Supplementary Figure 5(b), which does not display a clear signature of parafermion $\mathbb{Z}_3$ RR states for electrons. The number of states below the first entanglement gap is 11175, which diverges from the correct state counting of 9900. As demonstrated in the main manuscript, the relevant quantity in this case is the hole entanglement spectrum, which displays the right counting of quasi-particle excitations for the hole RR phase.

Supplementary References

[1] B. A. Bernevig and N. Regnault, Emergent many-body translational symmetries of Abelian and non-Abelian fractionally filled topological insulators, Phys. Rev. B **85**, 075128 (2012).

[2] F. D. M. Haldane, Fractional quantization of the Hall effect: A hierarchy of incompressible quantum fluid states, Phys. Rev. Lett. **51**, 605–608 (1983).

[3] B. I. Halperin, Statistics of quasiparticles and the hierarchy of fractional quantized Hall states, Phys. Rev. Lett. **52**, 1583–1586 (1984).

[4] J. K. Jain, Composite-fermion approach for the fractional quantum Hall effect, Phys. Rev. Lett. **63**, 199–202 (1989).

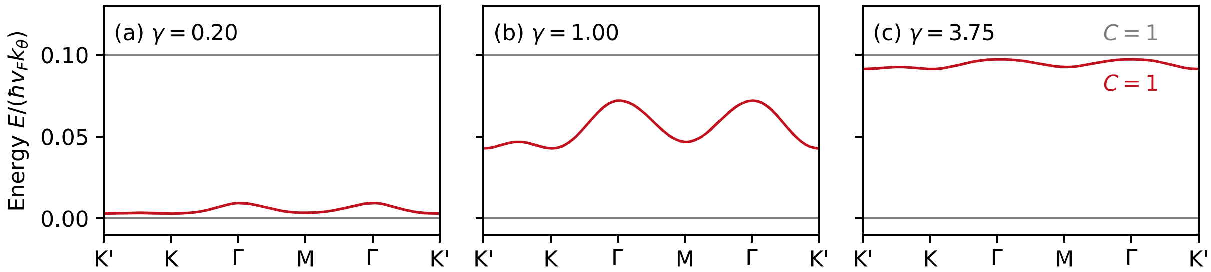

Supplementary Figure 1. Single-particle band structure. Topological nearly-flat bands at (a) $\gamma = 0.20$, (b) $\gamma = 1.00$ and (c) $\gamma = 3.75$ along the path $K' - K - \Gamma - \Gamma - K'$ in the moiré Brillouin zone. There are two degenerate flat bands at $E = 0$ and two non-degenerate bands (one flat and one nearly flat) with equal Chern number $\mathcal{C} = 1$ at positive energies. The band considered throughout this work is marked in red, while the other flat bands are marked in grey. The calculated energies are in units of $\hbar v_F k_\theta$ corresponding to the Hamiltonian introduced in the main manuscript (v_F is the Fermi velocity of graphene's Dirac cone).

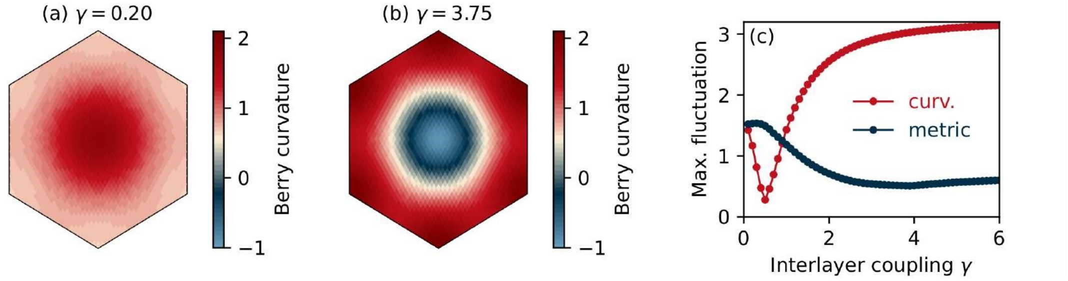

Supplementary Figure 2. Quantum geometry of the nearly flat band. Berry curvature distribution $\Omega(\mathbf{k})A_{\mathrm{BZ}}/2\pi$ in the mini Brillouin zone at (a) $\gamma = 0.20$ and (b) $\gamma = 3.75$. (c) Maximum fluctuation of the Berry curvature (red) and Fubini-Study metric (blue) as a function of γ. These calculations have been performed on a momentum grid of 40x40 points.

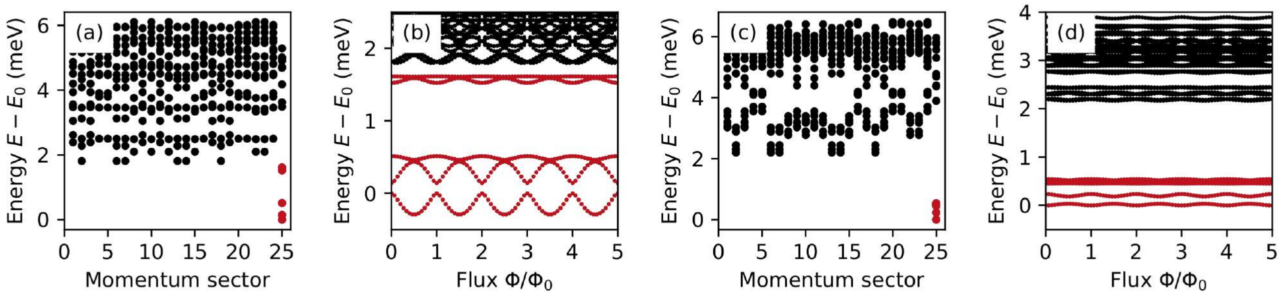

Supplementary Figure 3. Evidence for Abelian FCIs at weak coupling. (a) Low-lying many-body spectrum and (b) spectral flow at band filling $\nu = 3/5$ and $\gamma = 0.2$. The corresponding results for $\nu = 2/5$ are shown in (c)-(d). The fivefold quasi-degenerate ground states are labelled as red dots. Here, we employed a rectangular sample with $N_{\mathrm{s}} = 25$ sites.

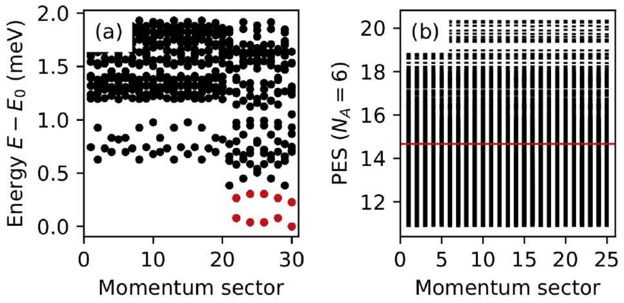

Supplementary Figure 4. Additional data for the $\nu = 3/5$ parafermion phase. (a) Many-body energy spectrum for a system size $N_{\mathrm{s}} = 30$ and (b) particle-cut entanglement spectrum ($N_A = 6$) for $N_{\mathrm{s}} = 25$. In both cases we have considered $\gamma = 3.75$. The red dots in (a) denote the quasi-degenerate Read–Rezayi ground states. The spanning vectors for the considered systems are $\mathbf{T}_1 = (5, 5)$ and $\mathbf{T}_2 = (-3, 3)$ for $N_{\mathrm{s}} = 30$ and $\mathbf{T}_1 = (5, 0)$ and $\mathbf{T}_2 = (0, 5)$ for $N_{\mathrm{s}} = 25$. The number of states below the red line in (b) is 160475, corresponding to the expected quasi-hole excitations for the Read–Rezayi phase.

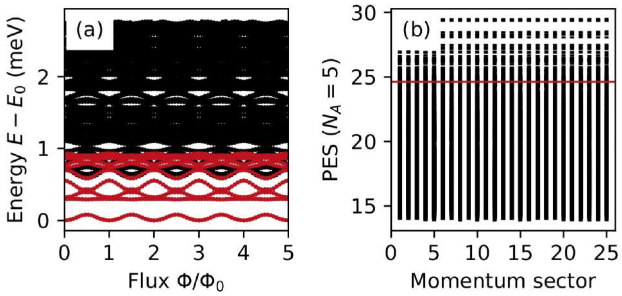

Supplementary Figure 5. Competing phases and quasi-hole excitations at $\nu = 2/5$. (a) Spectral flow and (b) particle-cut entanglement spectrum ($N_A = 5$) for $\gamma = 3.75$ and a system size $N_s = 25$. The red dots in (a) denote Read–Rezayi ground states. The number of states below the red line in (b) is 9900, corresponding to the expected quasi-hole excitations for the electron Read–Rezayi phase.
